# A large-area organic transistor with 3D-printed sensing gate for noninvasive single-molecule detection of pancreatic mucinous cyst markers

**DOI:** 10.1007/s00216-022-04040-4

**Published:** 2022-04-11

**Authors:** Lucia Sarcina, Fabrizio Viola, Francesco Modena, Rosaria Anna Picca, Paolo Bollella, Cinzia Di Franco, Nicola Cioffi, Mario Caironi, Ronald Österbacka, Irene Esposito, Gaetano Scamarcio, Luisa Torsi, Fabrizio Torricelli, Eleonora Macchia

**Affiliations:** 1 Dipartimento di Chimica, Università degli Studi di Bari Aldo Moro, Via E. Orabona 4, 70125 Bari, Italy; 2 Center for Nano Science and Technology@PoliMi, Istituto Italiano di Tecnologia, Via Pascoli 70/3, 20133 Milan, Italy; 3 Dipartimento di Elettronica, Infomazione e Bioingegneria; Politecnico di Milano, Milano, Italy; 4 CSGI (Centre for Colloid and Surface Science), Via E. Orabona 4, 70125 Bari, Italy; 5 The Faculty of Science and Engineering, Åbo Akademi University, Porthaninkatu 3, FI-20500 Turku, Finland; 6 Institute of Pathology, Heinrich-Heine University and University Hospital of Düsseldorf, 40225 Duesseldorf, Germany; 7 Dipartimento Interateneo di Fisica “M. Merlin”, Università degli Studi di Bari “Aldo Moro”, 70125 Bari, Italy; 8 Dipartimento Ingegneria dell’Informazione, Università degli Studi di Brescia, 25123 Brescia, Italy; 9 Dipartimento di Farmacia-Scienze del Farmaco, Università degli Studi di Bari Aldo Moro, Via E. Orabona 4, 70125 Bari, Italy

**Keywords:** Pancreatic cancer, Single-molecule assay, Inkjet-printed electronics, 3D-printed sensing gate module, Cost-effective bioelectronic platform

## Abstract

Early diagnosis in a premalignant (or pre-invasive) state represents the only chance for cure in neoplastic diseases such as pancreatic-biliary cancer, which are otherwise detected at later stages and can only be treated using palliative approaches, with no hope for a cure. Screening methods for the purpose of secondary prevention are not yet available for these cancers. Current diagnostic methods mostly rely on imaging techniques and conventional cytopathology, but they do not display adequate sensitivity to allow valid early diagnosis. Next-generation sequencing can be used to detect DNA markers down to the physical limit; however, this assay requires labeling and is time-consuming. The additional determination of a protein marker that is a predictor of aggressive behavior is a promising innovative approach, which holds the potential to improve diagnostic accuracy. Moreover, the possibility to detect biomarkers in blood serum offers the advantage of a noninvasive diagnosis. In this study, both the DNA and protein markers of pancreatic mucinous cysts were analyzed in human blood serum down to the single-molecule limit using the SiMoT (single-molecule assay with a large transistor) platform. The SiMoT device proposed herein, which exploits an inkjet-printed organic semiconductor on plastic foil, comprises an innovative 3D-printed sensing gate module, consisting of a truncated cone that protrudes from a plastic substrate and is compatible with standard ELISA wells. This 3D gate concept adds tremendous control over the biosensing system stability, along with minimal consumption of the capturing molecules and body fluid samples. The 3D sensing gate modules were extensively characterized from both a material and electrical perspective, successfully proving their suitability as detection interfaces for biosensing applications. KRAS and MUC1 target molecules were successfully analyzed in diluted human blood serum with the 3D sensing gate functionalized with b-KRAS and anti-MUC1, achieving a limit of detection of 10 zM and 40 zM, respectively. These limits of detection correspond to (1 ± 1) KRAS and (2 ± 1) MUC1 molecules in the 100 μL serum sample volume. This study provides a promising application of the 3D SiMoT platform, potentially facilitating the timely, noninvasive, and reliable identification of pancreatic cancer precursor cysts.

**Graphical abstract:**

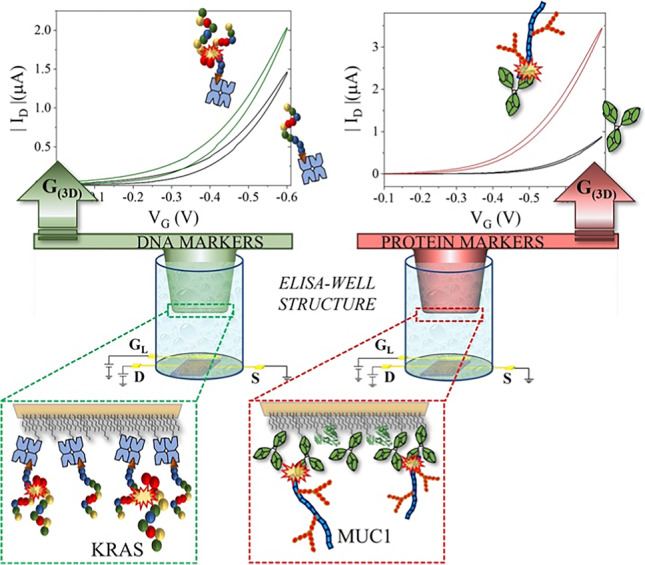

## Introduction

The electronic detection of clinically relevant biomarkers with single-molecule limit of detection (LOD) represents a new frontline to advance the field of precision medicine [1]. Enhancing the analytical performance of bioelectronic systems, including the ability to detect clinically relevant markers at the physical limit, may completely change the way healthcare is delivered. Indeed, bioelectronic systems will enable clinicians to associate the smallest biomarker variations to the individual pathological condition, already at its initial phase. Ultimately, clinicians will be able to track the exact moment of the initiation of the disease state, enabling precise medical treatments (i.e., precision medicine), which will result in decreased healthcare-associated costs. Currently, the social burden of pancreatic cancer in Europe is about one million life-years annually [2], and by 2030 it will be the second leading cause of cancer-related death in the Western world [3]. Surgical resection in very early stages currently represents the only potentially curative treatment option; however, 80–90% of patients receive a diagnosis already in advanced stages and only palliative therapies can be offered [4]. Therefore, identification of high-risk individuals and markers for early detection represents a crucial step in changing the course of this disease. In this regard, an ultra-sensitive assay can enable a cancer risk assessment, identifying early-stage cancers and discriminating between benign and malignant forms [5]. Mucinous cysts of the pancreas are precursors of pancreatic cancer [6]. However, they belong to the large and heterogeneous spectrum of cystic lesions. Despite numerous international efforts and guidelines aimed at delivering reliable criteria for the distinction between harmless and potentially dangerous cystic lesions in the presurgical setting [7, 8], differential diagnosis remains challenging. Recently, a few studies have reported the analysis of multiple DNA markers by next-generation sequencing (NGS), increasing the accuracy of cystic fluid analysis in the diagnosis and subclassification of pancreatic cysts [9]. Although the NGS method is capable of sequencing of DNA markers, such as KRAS, GNAS and TP53, with a LOD of a single molecule (copy), it is time-consuming, requiring up to 1 week from clinical sampling to result. Additionally, it is based exclusively on genomic alterations, which give important information about the type of cysts (i.e. non-neoplastic vs. neoplastic), but are much less reliable concerning the biological behavior (i.e. low vs. high risk). The addition of a protein marker that is a predictor of aggressive behavior is a promising innovative approach, which has been rarely investigated so far [10]. For instance, MUC1 is a glycoprotein almost exclusively expressed by high-risk pancreatic cysts as well as in cells of pancreatic cancer itself [11]. Therefore, the availability of a system enabling the detection of MUC1 at a single-molecule level in the context of a multiparametric analysis approach (for instance the detection of both protein and DNA markers) has the potential to increase further the diagnostic accuracy of high-risk precursors of pancreatic cancer. However, ultrasensitive protein detection remains an extremely challenging task. Indeed, Simoa^®^ technology can sense protein markers with attomolar (aM) LODs [12, 13], corresponding to 10^2^−10^3^ proteins in a sampled volume of 100 μL, being far less sensitive than NGS. Recently, Simoa Planar Array technology (SP-X System) [14] was developed based on a multiplex enzyme-linked immunosorbent assay (ELISA) system encompassing digital chemiluminescent imaging of an array of capture antibodies [15, 16]. Simoa SP-X technology can sense protein markers with LODs in the sub-femtomolar range. Moreover, Simoa and NGS are label-based, requiring laboratory facilities, and thus not at all suitable in point-of-care settings. Then, other relevant findings were accomplished, in which those technologies based on nanostructures seem to be the best candidates to consistently achieve label-free detection at the single-molecule level [17, 18]. They especially foresee the inspection of a femtoliter-sized volume in which the single-molecule under investigation is contained. The typical approach to single-molecule sensing, addressed as a near-field technique, entails a nanometer-sized detection interface. This implies a sensor component dimension comparable to that of the single-molecule to be detected, assuring a high signal-to-noise ratio [18].

Recently, the SiMoT (single-molecule assay with a large transistor) bioelectronic platform was successfully used for the detection of both proteins and DNA type markers, with a LOD at the single-molecule level [19–22]. This technology is based on an electrolyte-gated organic field-effect transistor (EG-OFET) [23], where a 0.5 cm^2^ gold gate electrode is biofunctionalized with ca. 10^12^ biorecognition elements, resulting in an extremely highly packed layer of capturing molecules. In this study, the SiMoT field-effect sensing system, involving a dual-gate structure, was fabricated by means of novel mass-producible, large-area-compatible, scalable techniques, including the inkjet printing of the organic semiconductor on plastic foil as well as the 3D printing of an innovative sensing gate module. By efficiently combining organic electronics and 3D printing processes, the 3D SiMoT platform was successfully used to assay KRAS and MUC1 mucinous lesions in blood serum samples with the same platform at the single-molecule detection limit. The platform is also single-use and cost-effective and can potentially work in low-resource settings. In particular, the 3D-printed sensing gate consists of a truncated cone that protrudes from a plastic substrate and is compatible with standard ELISA wells. The 3D gate is incubated in a standard ELISA plate containing the solutions involved in the biofunctionalization steps as well as in the human serum samples to be assayed. This approach provides a simple yet effective method for the biofunctionalization and incubation of the 3D gates with minimal consumption of the capturing molecules as well as body fluid samples. The amount of liquid for the biofunctionalization and incubation steps is the smallest possible, ensuring the cost-effectiveness of the biosensing system. Indeed, accounting for the nominal dimensions of both the ELISA plate well and the ad hoc design gate pillar, the maximum available volume is equal to 100 μL, while the minimum volume needed to enable the pillar top surface to touch the liquid is equal to 50 μL. Moreover, the design of the 3D SiMoT platform adds tremendous control over its stability, by combining a constant monitoring of the printed organic semiconductor operational stability in a water environment through the lateral reference gate and the fixed and optimized positioning of the 3D sensing gate. In addition, a protective layer of the connection tracks of the 3D sensing gate is designed in order to control the biofunctionalized gate area and to reduce spurious leakage currents as much as possible. Notably, a successful passivation strategy of tracks was proposed, leading to no contamination of the biofunctionalized gate area. The results gathered with the 3D SiMoT platform open the way for upscaling of the single biosensing device into an array structure manufactured using ultra-low-cost processes based on printed organic semiconductors and 3D printing, to obtain a multiplexing bioelectronic system. Moreover, the integration with the standard ELISA plates, used worldwide in clinical settings, can be foreseen, eventually delivering the fluids involved in the biofunctionalization and measurement phases by an automated procedure. Moreover, the lab-based device can be developed into a cost-effective portable prototype multiplexing array that integrates, with a modular approach, standard components and interfaces with novel materials, and it exhibits enhanced sensing capabilities for pancreatic cancer early diagnosis. Indeed, the 3D SiMoT will open the way for a massive use of high-throughput array-based assay not only in clinical laboratory analysis, but also in point-of-care and low-resource settings. The sensing platform is designed to be amenable for applications in several areas, and the final goal is to demonstrate that the detection of biomarkers at ultra-low levels holds the potential to enable new options for diagnostics and treatment of progressive diseases. The SiMoT platform endowed with a 3D gate component represents a very powerful technology to enable noninvasive early diagnosis of life-threatening progressive diseases. In particular, new diagnostic methods based on assay of the markers entirely in a peripheral fluid, with extremely low detection limits, should be of great relevance to replace the much more invasive procedures currently performed by means of biopsy. Such an analytical tool will indeed enable clinicians to associate the tiniest increase in a biomarker with the progression of a disease, particularly at its early stage. Eventually, the onset of the illness can be determined by physicians with higher accuracy. This will greatly enhance their ability to cure diseases by supporting better prognosis and permitting the application of precise treatment methods. An enormous improvement in the quality of life and longevity of the population for generations to come is foreseen along with a reduction in healthcare expenses.

## Materials and methods

### Materials

Bovine serum albumin (BSA), high-performance liquid chromatography (HPLC)-grade water, high-grade ethanol (p.a. assay ≥ 99.8 %), streptavidin (SAV) from *Streptomyces avidinii*, phosphate-buffered saline (PBS) tablets, poly(3-hexylthiophene) (P3HT, high regioregularity ≥ 99%, average molecular weight [M_W_] of 20–45 kDa) were purchased from Sigma-Aldrich and used without any further purification. The device components, namely lateral gate and interdigitated source-drain electrodes, were printed on a 125 μm-thick poly(ethylene 2,6-naphthalate) (PEN) substrate, obtained from DuPont. The mucin 1 bioreceptor (anti-MUC1, clone UMAB57, code: UM800008) and the recombinant protein of human mucin 1, cell surface-associated (MUC1), transcript variant 2 (MUC1, M_W_ = 25,100 Da, product number: TP321390) were provided by OriGene. 3-Mercaptopropionic acid (3-MPA), 11-mercaptoundecanoic acid (11-MUA), 1-ethyl-3-(3-dimethylaminopropyl) carbodiimide (EDC) and the *N*-hydroxysulfosuccinimide sodium salt (Sulfo-NHS) were obtained from Merck Millipore (now MilliporeSigma). KRAS G12D (product number C9745(D07)) as target oligonucleotide, a biotinylated-KRAS G12D fwd (b-KRAS) (product number C9745(E03)) as biorecognition element and KRAS G12D 1MM (product number C9745(D12)) (one mismatch in correspondence of 14th nucleic acid base, used as negative control) were provided by Invitrogen-Thermo Fisher Scientific (oligonucleotides sequences are specified in previous reports) [10]. Human serum samples were obtained from blood samples collected from different healthy donor (age range 18–60 years, Research Donors Ltd of London [UK]) and diluted 1:4 with PBS and successively centrifuged at 10,000×*g* for 5 min, before the 3D SiMoT assay.

### Electrolyte-gated organic thin-film transistor fabrication

EG-OFETs with an inkjet-printed organic semiconductor were prepared on a flexible and PEN substrate as previously reported [24]. Afterwards, the samples were cleaned in acetone and 2-propanol (IPA) under ultrasonication, gently dried with a nitrogen flow and additionally cleaned for 2 min by means of oxygen plasma. S and D electrodes were photolithographically defined, possessing a 10.5 mm (width) × 5 μm (length) channel, while the circular lateral gate (LG) exhibited a radius of 1.25 mm. P3HT (2.6 mg/mL) was dissolved of in a mixture of chlorobenzene (CB) and *o*-dichlorobenzene (ODCB), 75:25 v/v, and inkjet-printed with a Fujifilm Dimatix DMP-2831. Details on the printed EG-OFET fabrication and operational stability in a water environment have been reported elsewhere [24].

### Fabrication and characterization of the 3D-printed sensing gate

**Fig. 1:**
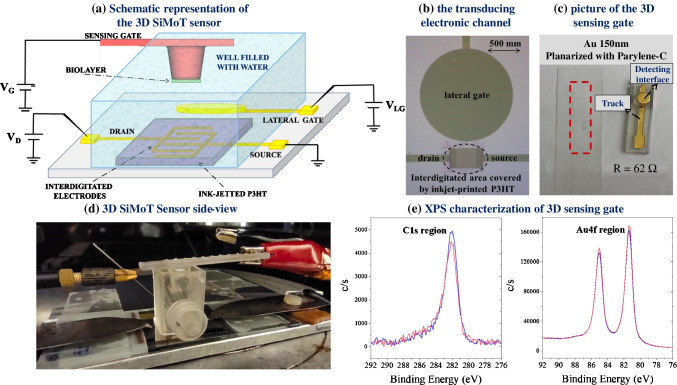
(**a**) A schematic of the 3D SiMoT biosensing platform. (**b**) Micrograph of the transduction electronic channel encompassing the inkjet-printed P3HT deposited on top of the interdigitated source and drain electrodes along with the reference lateral gate. (**c**) Picture of the 3D-printed sensing gate, along with the “scotch test” highlighted in the red dashed box. (**d**) Side view of the 3D SiMoT sensor. (**e**) C1s (left) and Au4f (right) regions relevant to detection interface of the 3D sensing gate with Parylene-C passivation (red curves) and without passivation (blue curves) of the track

The 3D gate structure consists of a hollow pillar (protruding 6.5 mm with a 0.3 mm-thick cavity to avoid metal interconnections with the ELISA plate surface) as shown in Fig. 1c. The square surface (used as gate pad) is 9 × 9 × 1.5 mm. The top base radius of the pillar is 2.8 mm, while the bottom base radius is 3.25 mm, and a side wall slope of 3.9°. The proposed sensing platform was conceived and optimized to fit into a commercial ELISA plate during its biofunctionalization, as reported in the next section. Hence, the metal interconnection passes through a slit, without touching the ELISA plate. The fabrication of the 3D structures was performed by using additive printing techniques. To this aim, a 3D printing stereolithography (SLA) approach was selected because it combines all the benefits of the 3D printing techniques such as fast and flexible design, no waste of material and low cost, with the high accuracy, fine features and wide range of materials suitable for a large set of experimental conditions and applications. A Form 2 printer was used, encompassing the following specifications: layer thickness = 100 μm; raft type, full raft; density, 1.20; touchpoint size, 0.45 mm; height above rafts, 10.00; raft thickness, 2.00 mm; tilt angle α_x_ = α_y_ = 45°. Clear Resin FLGPCL04 was used as the material, enabling high-resolution rapid prototyping with excellent solvent compatibility, and good temperature properties with a heat deflection temperature greater than 73°C after the post-process annealing and UV curing. For the post-curing, the samples are inserted in a UV oven with a hotplate set at 65 °C for 20 min for warming, and the UV light is then turned on for 30 min while keeping the hotplate on. The preheating step enables the printed samples to reach a uniform temperature before the UV exposure. The curing rate of the resin depends on its temperature, and the more uniform the temperature, the more uniform the curing of the resin, and the lower the residual stress due to the uneven curing of the sample. The 3D gate roughness was reduced prior to the evaporation process by depositing a layer of Parylene-C of 2 μm thickness. Parylene refers to the category of chemical vapor-deposited poly(p-xylylene) polymers, proposed as insulating layers. Among them, Parylene-C is the most widely used, as it combines excellent barrier properties and cost-effectiveness, along with other processing benefits. Indeed, Parylene-C was selected because it is an excellent dielectric, transparent, with good mechanical properties that can be deposited by large-area industrial processes. It also has constant coating thickness independent of the substrate geometry, it is pinhole-free, it is biocompatible, and it has excellent chemical properties, being stable and inert with a large range of solvents, including those used for the gate biofunctionalization (i.e. ethanol, PBS and water). Parylene-C was deposited through chemical vapor deposition (CVD) with a Labcoter PDS 2010 (Specialty Coating Systems, SCS). The 3D gates were inserted in the deposition chamber, and 10 g of Parylene-C in the form of solid dimer was placed inside the vaporization chamber. Then, the temperature inside the vaporization chamber was increased to 175°C. The dimer begins to sublimate reaching the pyrolysis chamber, where the pressure is kept at 0.5 torr and the temperature at 690°C. These conditions convert the dimer to a monomer phase. Once reaching the polymerization chamber (kept at RT and 0.01 torr), the monomers begin the process of polymerization by forming covalent bonds. Eventually, the polymeric chains become long enough to bind to the substrate under the effect of gravity. After the planarization step, gold was thermally evaporated directly on the plastic structure with Parylene-C. The gold thickness was about 150 nm. Subsequently, Parylene-C was also employed as a protective layer of the tracks using the very same protocol as involved in the gate planarization step. To prevent the deposition of the Parylene-C passivation layer over the detection interface of the 3D sensing gates, the circular pads were covered by sticky tape before the passivation step, while the connection tracks were not covered. The result of the process was a conformal coating of Parylene-C on the samples, with a thickness of 5 μm which covered only the connection tracks of the 3D gates. After the deposition, the 3D gates with passivated tracks were washed with IPA and then cleaned with a plasma oxygen treatment for 120 s (O_2_ pressure 0.4 mbar, power = 100 W, Femto Plasma Asher, Diener Electronic GmbH & Co. KG). In addition, 3D gates, with or without track passivation with Parylene, were characterized by X-ray photoelectron spectroscopy (XPS). To this aim, both the detection interfaces and tracks were characterized and the identified elements were quantified. The samples were not cleaned before analysis and were cut with a surgical knife to be loaded in the spectrometer. Samples were analyzed using a PHI Versaprobe II Spectrometer. Monochromated Al Kα radiation (1486.6 eV) was used, with a spot size of 200 μm. Survey spectra were acquired with a pass energy of 117.4 eV, while high-resolution (HR) spectra were acquired with a pass energy of 58.7 eV. C1s, O1s, Au4f, and Cl2p regions were investigated. MultiPak™ (v. 9.9.0.8, PHI-ULVAC) software was used to process the data. Binding energy (BE) scale was corrected taking as reference the C1s component at 284.8 eV.

### Gate biofunctionalization protocol

The 3D sensing gate modules were thoroughly washed using an ultrasonic bath in IPA for 10 min and subsequently rinsed with HPLC water, dried under N_2_ flux and then treated for 2 min in an ozone cleaner. The gate biofunctionalization protocol has been optimized and described in details elsewhere [23, 25]. Briefly, it undergoes the following steps: (i) 10 mM chemical self-assembled monolayer (chem-SAM) of 3-MPA to 11-MUA, with 10:1 molar ratio in ethanol for 18 h, (ii) chemical SAM (chem-SAM) activation with EDC (200 mM)/NHS (50 mM) for 2 h, (iii a) the single-strand bioprobe (oligonucleotide, b-KRAS) was immobilized through reaction with SAV (1.5 μM) for 2 h, deactivated with ethanolamine (1 M) and further reacted with biotinylated KRAS (0.5 μM) for 2 h, while for the anti-MUC1 (iii b) the 3D gate (with activated chem-SAM) was directly incubated with 0.4 mM (10 μg mL^−1^) of anti-MUC1, then deactivated with ethanolamine (1 M) and reacted with 1.5 μM (0.1 mg mL^−1^) BSA to prevent nonspecific binding. For anti-MUC1, the control experiment was performed by using a gate biofunctionalized only with BSA (0.1 mg mL^−1^). Moreover, for KRAS, KRAS G12D 1MM (one mismatch in correspondence of 14th nucleic acid base, used ass negative control) was used as control experiment. The whole biofunctionalization protocol was performed on a standard ELISA plate, using a volume of 100 μL of solution of each reagent. The gate biofunctionalization protocol was monitored through surface plasmon resonance (BioNavis multi-parameter surface plasmon resonance (MP-SPR) Navi™ 200) reproducing the biofunctionalization protocol on a glass slide coated with a 50 nm gold layer.

### Sensing measurements

The 3D SiMoT device with an inkjet-printed organic semiconductor and lateral gate on plastic foil was stabilized before performing sensing experiments, according to the protocol defined and validated elsewhere [10, 24]. The sensing measurements were performed according to the following protocol. Each 3D sensing gate, for either DNA and protein assay, is washed thoroughly with HPLC water and mounted on top of the EG-OFET cell. A set of 20 transfer characteristics were recorded by sweeping the gate voltage V_G_ from 0.1 V to −0.5 V and keeping fixed the drain voltage V_D_ (−0.3 V) (each curve measured with a delay of 10 s). Next, the sensing gate was incubated for 10 min in 100 μl of human serum diluted 1:4. After the incubation, the functionalized 3D gate was thoroughly washed with HPLC water, and a new set of *I–V* transfer curves were registered. After *I*_0_ baseline measurement, the sensing gate was incubated in 100 μl of the human serum solutions spiked with standard aliquots of KRAS G12D rev or MUC1 ligands at concentrations ranging from 10 zM (1 × 10^−20^ M) to 1 fM (1 × 10^−15^ M) for 10 min. After incubation in each of the KRAS G12D rev or MUC1 sample, a further set of *I–V* transfer curves were measured, thus providing the signal *I*. The KRAS G12D rev and MUC1 standard solutions in diluted human blood serum were prepared by a serial dilution process according to the following equation: c_2_ = c_1_ · V_1_ / V_2_ =k · c_1_. Here, c_1_ and c_2_ are the ligand concentrations in the stock and in the diluted solution, respectively, while V_1_ and V_2_ are the corresponding solution volumes and k = V_1_ / V_2_ is the dilution factor. As is customary, the former dilution is the stock solution for the subsequent dilution in the series. Standard tenfold serial dilutions were performed starting from concentrated analyte mother solutions. The mother solutions have a concentration of 40 μM for the KRAS G12D target oligonucleotide and 100 nM for MUC1. The absolute uncertainty of the concentration for each standard solution was computed as the propagation error of the dilution factor, while the uncertainty of the volume, given by the supplier company of the pipettes used, is 1%. This value of the uncertainty of the volume considers both random and systematic errors in pipetting. The nominal number of MUC1 or KRAS (#Ligand) at each concentration is given by cVN_A_, where c is the ligand concentration, V is the volume of the standard PBS solution in which the gate is incubated (100 μL) and N_A_ is the Avogadro number. Indeed, the 3D sensing gate was incubated into an aliquot of 100 μL of the ligand standard solution, as customary in single-molecule wide-field detection techniques [12, 26]. The error associated with the sampling procedure can be estimated according to the Poisson distribution. The total uncertainty of the ligand concentration was evaluated as the square root of the sum of the squares (RSS) of the dilution (σ_D_) and Poisson (σ_P_) errors. Therefore, the sensing gate was incubated in the human serum solutions hosting in 100 μl KRAS G12D rev or MUC1 ligands ranging from 1 ± 1 molecules (10 zM) to (6 10^4^ ± 2 10^2^) molecules (1 fM). In large-area (wide-field) transduction interfaces, a higher active area is exposed compared to near-field biosensing methods, thus proving to be a viable solution to overcome the diffusion barrier issue. This evidence is well supported by several experimental studies, mostly considering large-area interface field-effect transistors (FET) [27–29]. In addition, the latter exhibited LOD down to the attomolar level (aM, 10^−18^ M), with a response time shorter than hundreds of seconds. As demonstrated by several research groups [26], a single molecule in 100 μl (concentration of ~ 10–20 zM) undergoes diffusion and eventually impinging at millimeter-wide surface populated with trillions of recognition elements within a timescale of minutes. It was recently demonstrated how at least one out of a few molecules (< 10) diffusing in a large volume can impinge within 10 min on the large interface, generating a detectable signal at the LOD [30]. Importantly, the model also reveals that the fast spinning of the diffusing ligand enables us to quickly find the right orientation to bind to one capturing element independently of its orientation. Therefore, a FET bioelectronic sensor comprising a micrometric- or a millimetric-wide detection interface can perform single-molecule detection, being unaffected by diffusion-barrier issues, in contrast to nanometric interfaces. During each incubation step, the reference gate was monitored registering five transfer characteristics, each one with a delay of 10 s in the same voltage window utilized for the sensing gate. As negative control experiments, the following dose curves were registered for the (i) DNA and (ii) protein assays, respectively. The negative control experiments were designed, in the case of the DNA assay (i), with the dose curves involving the detection of KRAS with a single mismatch in the 14th base, against the same biotinylated probe attached to the gate, while for the protein assay, (ii) the dose curves involve the detection of MUC1 against a sensing gate biofunctionalized with BSA (0.1 mg mL^−1^). All the incubation steps were performed using a standard ELISA plate with incubation volume of 100 μL. Remarkably, a non-regenerative approach has been undertaken [31], due to the irreversible nature of the biorecognition element/ligand binding. Indeed, gold gate surface regeneration between consecutive analyte injections to remove the bound analytes cannot be used in case of stable ligand-analyte complexes, such as biorecognition element-analyte binding interactions involved in this study. In fact, with such systems the regeneration may fail in removing all the bound analyte molecules. As a result, the 3D SiMoT signal would not be close enough to the baseline after the regeneration. All data were plotted and analyzed with Origin2018.

## Results and discussion

The 3D SiMoT biosensing platform proposed in this study is outlined in Fig. 1. The device structure shown in Fig. 1a is based on the cost-effective EG-OFET, with inkjet-printed P3HT as channel material on top of the interdigitated source (S) and drain (D) electrodes, as shown in Fig. 1b.

The sensor consists of reference and sensing gate modules that are submerged, along with the FET area, in the water electrolyte (HPLC-grade). The reference coplanar gate (LG) (Fig. 1a and b) opportunely operates as an internal reference electrode facilitating continuous monitoring of the EG-OFET current level, or in other words, the device operational stability. In Fig. 1b, a micrograph of the patterned source and drain interdigitated gold electrodes and of the circular lateral gate is shown. The top gate module, namely the 3D sensing gate, is a 3D-printed structure and is made of a gold thin film (Au thickness 150 nm) deposited by thermal evaporation through a 3D-printed shadow mask on a plastic substrate planarized with Parylene-C, as shown in Fig. 1c. According to the ad hoc developed design of the 3D-printed sensing module, when the pillars are inserted in the ELISA plate, the distance between the bottom of the ELISA plate and the top surface of the pillar is 3.3 mm. It should be noted that the total ELISA well height is 9.8 mm and the height of the cone bottom is 6.5 mm. As a consequence, accounting for the nominal dimensions of both the ELISA plate hole and the gate pillar, the maximum available volume results 101.12 μL, while the minimum volume enabling the pillar top surface to touch the liquid is 52.72 μL. Taking into account a minimum printing process tolerance of ± 100 μm, the minimum amount of liquid for the incubation phase results 60 μL. To be conservative, a maximum variability of the pillar height equal to 3× printing process tolerance, i.e. ±300 μm, can be considered, and hence an incubation volume of 65 μL is required. Such volume compensates for the process variability while still minimizing both the maximum uncertainty of the sample volume [23], being lower than 0.2 %, and the biofunctionalization cost. To assess the adhesion of the evaporated gold on the printed substrate the “scotch test” was performed. On the basis of the protocol developed elsewhere [32], 3M Scotch Magic Tape is accurately stuck onto the sample and then quickly peeled away. The test is passed when no gold is transferred to the tape, as for example displayed in Fig. 1c inside the red dashed box. Therefore, the evaporated gold has an excellent adhesion to the substrate. In addition, the total electrical resistance of the track is reported in Fig. 1c as well. An average resistance value of (60 ± 8) Ω was estimated on the 15 3D gates characterized and used in this study. Based on those results, it can be inferred that both the good quality of Au gates and low resistance of the tracks can be obtained by depositing an Au layer with a thickness equal to 150 nm directly on the planarized 3D-printed plastic substrate. Figure 1d shows a picture of the side-view of the sensor. As a further step in the 3D sensing gate characterization, the evaluation of the track passivation with a Parylene-C protective layer deposited through chemical vapor deposition (CVD) was assessed. To this aim, 3D gates with and without track passivation with Parylene-C were studied by X-ray photoelectron spectroscopy (XPS) to evaluate potential contamination of the gate detection interface. In particular, both the detection interface areas and tracks were characterized and the identified elements were quantified. Spectra for each representative area (gate or track) for both types of electrodes were registered to estimate the chemical surface composition. First, survey scans in different areas were collected to identify the main elements. The detection interface and track for the non-passivated electrode present signals relevant to gold, carbon, and oxygen whereas carbon, chlorine, and oxygen are detected for the Parylene-passivated track. Typical surface chemical composition for detection interfaces and tracks is reported in Table 1. It is clear that contamination of the gold detection interface with Parylene-C residues does not occur, as chlorine (discriminant element for Parylene-C) is present as trace. Moreover, passivation of the tracks was successful.

**Table 1.** Typical surface chemical composition of analyzed areas of 3D gates. Errors are determined on three replicates

Area	C%	O%	Au%	Cl%
Detection interface (with passivation)	36 ± 4	14 ± 2	50 ± 5	≤ 0.5
Detection interface (w/o passivation)	36 ± 3	12 ± 2	52 ± 3	–
Track (with passivation)	80 ± 2	16 ± 3	≤ 0.2	4.0 ± 0.5
Track (w/o passivation)	55 ± 5	12 ± 2	33 ± 3	–

**Fig. 2:**
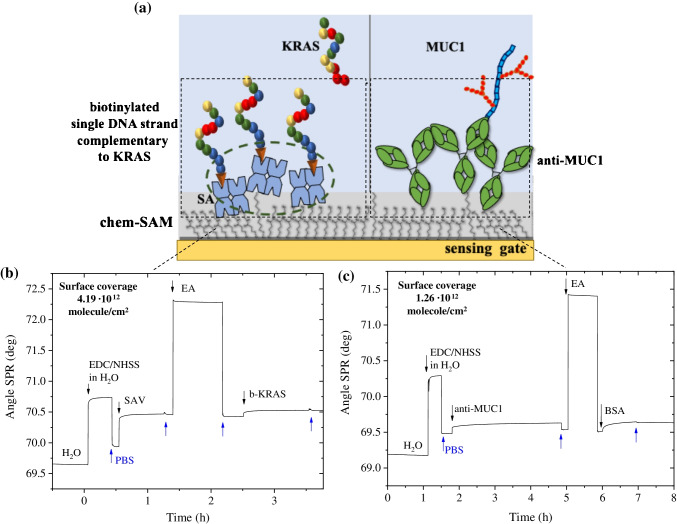
(**a**) Pictorial view of the b-KRAS and anti-MUC1 bio-SAM layers attached to the chem-SAM. Surface plasmon resonance traces to quantify the immobilized b-KRAS (**b**) and anti-MUC1 (**c**). The SPR traces of the immobilization of the b-KRAS and anti-MUC1 on the Au surface are modified with the mixed alkanethiol chem-SAM

Additional evidence that the gate detection interface of the 3D electrodes treated with Parylene-C is not contaminated or modified can be found comparing C1s and Au4f regions of two gate detection interface areas, reported in Fig. 1e, one belonging to a sample that did not undergo the passivation protocol, shown as blue line, and the other belonging to a sample that was exposed to the passivation protocol, shown as a red line. It is evident that the two photoelectronic signals are almost overlapping. The 3D sensing gate surface with passivated tracks was further biofunctionalized with the pancreatic mucinous cyst biomarkers, according to the protocol described in previous studies [22, 23, 33]. The key component of the 3D SiMoT sensor relies on the biofunctionalized bio-SAM grown onto the gold surface of the 3D detection interface. The latter allows the device to achieve the selective recognition of the biomarker of interest. In this study two biofunctionalization protocols were tested: one involving b-KRAS, being a biotinylated single-strand DNA possessing a gene sequence complementary to that of KRAS, while the other involves the anti-MUC1, serving as capturing antibodies for the MUC1. These two biofunctionalization approaches are outlined in Fig. 2a. The in situ and operando characterization of both biofunctionalization protocols was performed via a surface plasmon resonance (SPR) apparatus, in the so-called Kretschmann configuration [34]. The surface plasmon generation mechanism relies on the presence of a thin film of a noble metal (e.g., 50 nm gold layer), capable of partial loss of the energy of the reflected light, which is spent by the excitation of metal surface electrons. This determines the generation of an evanescent wave that propagates along the interface between the metal layer and the dielectric material (sample medium) [35]. This plasma wave is mainly restrained at the metal–dielectric interface, resulting in a higher concentration of the field in the dielectric while decreasing exponentially in the bulk of both materials [36]. Hence, the technique is sensitive to any change occurring within the first 300–400 nm of the gold facing the dielectric [37]. Here the local refractive index variations are correlated with the biomolecule interactions that can be inspected. The SPR apparatus used for this work was arranged so that the gold-exposed area, about 0.4 cm^2^, could be simultaneously inspected in two different points (3 mm apart), to assess the thickness uniformity of the deposited layer. According to the SAM protocol, the gold surface was functionalized with the mixed chem-SAM prior to the SPR characterization. The modified gold slide was allotted into the sample-holder, facing the SPR flow-through cell. Then, all bio-SAM immobilization was performed by static injection of 300 μL at 22 °C of each solution involved in the biofunctionalization protocol and monitored in real time. Figure 2b and c shows the typical sensograms obtained during all bio-conjugation steps of the bio-SAM layer for b-KRAS and anti-MUC1, respectively.

It is possible to compute the amount of b-KRAS and anti-MUC1 deposited per unit area from the layer thickness and the refractive index variation of non-homogeneous protein deposited on the sensor surface by using the de Feijter equation:

1 $${\Gamma =\frac{\left({n}_a-{n}_m\right){d}_a}{\frac{dn}{dc}}}$$

where *n*_a_ and *n*_m_ are the average refractive index of the biolayer and the refractive index of the buffer solution, respectively, *d*_a_ is the average thickness of the biolayer, and *dn/dc* is the specific refractivity associated to the biolayer. By including the instrument signal in Eq. (1), the following equation holds true:

2 $$\left({n}_a-{n}_m\right)=\varDelta \theta \ast k$$

where *k* is a coefficient for sensitivity (wavelength-dependent), and *Δθ* is the measured angular response (angle shift) in the measurement. Therefore, Eq. (1) becomes

3 $$\Gamma =\frac{\Delta \theta \ast k\ast {d}_a}{\frac{dn}{dc}}$$

For a thin layer (100 nm maximum thickness), the product k_*_d_*a*_ can be approximated to be constant and equal to 1.0 × 10^−7^ cm/deg for the laser wavelength used in this study (λ = 670 nm). Following the literature, the *dn/dc* value can be approximated to 0.182 cm^3^/g at 670 nm [38], leading to the calculation of biomolecule surface coverage as

4 $$\Gamma =\Delta \theta \ast 550\ ng/{cm}^2$$

Upon injection of 100 μL of a 0.5 μM b-KRAS buffer solution, an angular shift is registered, as seen in Fig. 2b. Using Eq. (4), the resulting surface coverage Γ = 52.7 ± 0.1 ng/cm^2^ is calculated at equilibrium after removing the b-KRAS excess. By considering the molecular weight of a single b-KRAS strand, the average number of active binding sites available is estimated at about (4.01 ± 0.01) × 10^12^ molecules/cm^2^. Similarly, when 10 μg mL^−1^ of anti-MUC1 buffer solution is injected, the resulting surface coverage is equal to Γ = 52 ± 1 ng/cm^2^, corresponding to an estimated average total number of binding sites effectively available of (2.10 ± 0.05) × 10^11^ molecules/cm^2^. This is a very densely packed recognition layer, as customary for a SiMoT device. Moreover, the covalent approach used herein in the anti-MUC1 immobilization onto the gate surface modified with the chem-SAM occurs via available functional groups of exposed amino acids. Remarkably, protein exhibits random orientation upon covalent binding due to the immobilization through –NH_2_ groups available at several amino acid residues (e.g., lysine), resulting in a heterogeneous and non-oriented biofilm. However, the SPR surface characterization, demonstrating the availability of ~10^12^ bioactive capturing anti-MUC1, along with the SAM surface characterization [23] strongly suggests that the anti-MUC1 packed in the SAM largely lay edge-on with one of their Fab fragments pointing away from the gate surface. Therefore, the resulting layer is probably characterized by a certain degree of order [39]. Moreover, it is reported that, at physiological pH, antibodies bear a dipole moment oriented from the Fc to the Fab region, and thus the overall weakly oriented dipoles are likely to provide the bio-SAM with electrostatic properties. Such evidence can also possibly add a weak electrostatic driving force component to the affinity binding process.

**Fig. 3.**
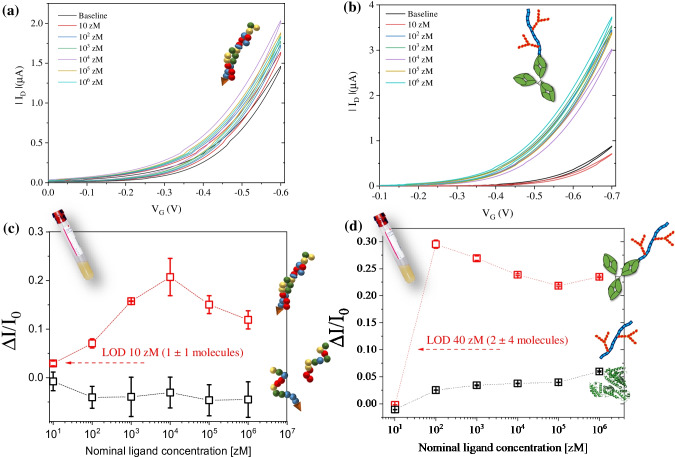
Stable transfer characteristics (I_D_ vs. V_G_ at fixed V_D_ = −0.3V) upon exposure to diluted human blood serum, as the baseline signal, and further exposed to human blood serum spiked with (**a**) KRAS and (**b**) MUC1 with concentrations ranging from 10 zM to 1 fM. (**c**) KRAS/b-KRAS dose–response curve (hollow red squares) are presented as the ΔI/I_0_ vs. KRAS concentration. The negative control experiment towards KRAS with one mismatch is reported as hollow black squares. (**d**) MUC1/anti-MUC1 dose–response curve (hollow red squares) are presented as the ΔI/I_0_ vs. MUC1 concentration. A BSA biofunctionalized gate was employed in the control experiment (hollow black squares). All the data points are provided as the average of at least three replicates, while the error bars were computed as the relative standard deviation, providing an estimation of the reproducibility of the assay

The sensing experiments encompass the measurement of a set of 20 transfer characteristics with the 3D SiMoT biosensing platform, each one registered with a delay of 10 s sweeping V_G_ in the range of 0.1 V to −0.5 V and keeping V_D_ fixed at −0.3 V. The sensing gate can be incubated at room temperature for 10 min in 100 μL of human blood serum diluted 1:4. The functionalized gate is removed from the serum solution and washed thoroughly with HPLC water, and a new set of transfer characteristics is registered. When a stable *I*_0_ baseline has been registered, the same gate is immersed and incubated for 10 min in 100 μL of the serum standard solutions with the target ligands at concentrations equal to 10 zM (1 × 10^−20^ M), 100 zM (1 × 10^−19^ M), 1 aM (1 × 10^−18^ M), 10 aM (1 × 10^−17^ M), 100 aM (1 × 10^−16^ M), and 1 fM (1 × 10^−15^ M). After incubation in each of the serum solutions spiked with the relevant ligand beginning from the least concentrated, the sensing gate is carefully washed with water (HPLC grade). The sensing gate is then placed in the well of the sensing device, and subsequently a group of *I–V* transfer curves is recorded. The final and stable I_D_–V_G_ curves at each concentration for both the KRAS and MUC1 sensing are reported in Fig. 3a and b, respectively. As apparent from the inspection of the transfer characteristics, a negligible hysteresis, evaluated as the difference between source-drain current values measured in the forward and backward gate voltage sweep [40], was registered, being at most 6%. Figure 3c and d shows the relative current changes, namely ΔI/I_0_ being equal to $\frac{I-{I}_0}{I_0}$, as a function of the nominal ligand concentration (hollow red squares), for the genomic and protein assays, respectively. Remarkably, the baseline and signal currents used to evaluate the ΔI/I_0_ were taken at maximum V_G_, where the minimum hysteresis of transfer characteristic was registered, being always below 1%. The error bars were computed as the relative standard deviation evaluated with at least three different 3D-printed functionalized gates. Negative control experiments were also registered for both the assays, performing ad hoc designed experiments. As far as the DNA-based assay is concerned, the negative control dose curves involved the detection of an oligonucleotide sequence identical to that of KRAS possessing a single mismatch in the 14th base. This was assayed against the same b-KRAS biotinylated probe giving a nonbinding response. The data are displayed in Fig. 3c as hollow black squares. On the other hand, negative control dose curves involved the detection of MUC1 against a 3D sensing electrode biofunctionalized with a nonbinding protein such as BSA. The data are displayed in Fig. 3d as hollow black squares. As can be seen from the data for the negative control experiments, the responses are indeed zero for both assays. Notably, the negative control dose curves did not show any significant signal, clearly confirming the selectivity of the 3D SiMoT platform against both genetic and protein markers. The evaluation of the dose curves reported in Fig. 3c and d customarily also displays a large variation of I_D_, which is registered already at concentrations as low as 10 zM of KRAS and 100 zM of MUC1. All the data points are provided as the average of at least three replicates, while the error bars have been computed as the relative standard deviation, providing an estimation of the reproducibility of the assay, being within 5% at most. The repeatability evaluated over five subsequent transfer characteristics is customarily within 2%.

According to the IUPAC definition, the LOD was estimated as the concentration corresponding to a response equal to (*ΔI/I*_*0*_)_*mean*_ *± kσ*, where (*ΔI/I*_*0*_)_*mean*_ is the average response of the negative control experiment, while *σ* is its standard deviation and k is selected depending on the requested confidence level [41]. IUPAC recommends to select k equal to 3, resulting in the probability of a negative control signal being threefold higher than the (*ΔI/I*_*0*_)_*mean*_ (i.e. a false positive being less than 1%). Considering the noise level and the standard deviation of the control experiments of each assay, LOD levels of −4% and −10 % were estimated for KRAS and MUC1 assays, respectively. Therefore, the LOD computed for the KRAS assay is equal to 10 zM, corresponding to 1 ± 1 molecule in the 100 μL sampled volume. Moreover, the LOD level matches to MUC1 nominal concentration of 40 zM, corresponding to 2 ± 1 molecules in the 100 μL sampled volume. As it is apparent all the assays, in diluted blood serum, detect at the physical limit both proteins and DNA markers using the same 3D SiMoT platform.

Remarkably, the sigmoid exponential dependence from the analyte concentration reported in Fig. 3c and d suggests a binary response, capable of assessing the total absence or the presence of just a single molecule of a marker of a progressive disease in the sampled biofluid. The amplification mechanism at the basis of the ultra-high sensitivity of the 3D SiMoT platform might be enabled by a built-in mechanism that switches the gate biolayer work function, measured as a shift of the SiMoT threshold voltage (V_T_). Such a mechanism has been postulated and is described in detail elsewhere and briefly recalled [22, 23]. The amplification mechanism is assumed to rely on an electrostatic network of dipoles (hydrogen bonding) connecting the packed molecules of the biolayer. Operating the device in the gate field orients these dipoles, while the binding event triggers a conformational change that generates a defect, changing the local dipole arrangement (work function) that propagates in the gating field, amplifying the single-binding signal. The main features of this biolayer amplification effect is that an up to 30% relative output current change compared to the background signal is registered when only few analyte molecules are in the biofluid solution, while the background noise, evaluated on a negative control experiment, is only within 5% of the relative standard deviation. Therefore, this platform might be of enormous importance in supporting clinicians to sort an asymptomatic population into those who have been tested with at least one marker, likely to be diseased, from those who have no marker, likely to be healthy.

## Conclusions

In conclusion, in this study the design, fabrication process flow and the development of a 3D SiMoT biosensing system have been reported. Remarkably, the 3D SiMoT device has been proven capable of detecting at the single-molecule level both KRAS and MUC1 markers in human blood serum. The validation of the 3D-printed sensing gate module was carried out by measuring the transfer characteristic curves of an EG-OFET device comprising an inkjet-printed organic semiconductor and a lateral gate on plastic foil. In particular, the adopted 3D printing technology and the design flow for the realization of the sensing gates were optimized, enabling minimization of the volume of biofluids used for the biofunctionalization of the gate electrodes as well as for the assays, while maintaining full compatibility with the standard ELISA plate. The 3D sensing gate modules were extensively characterized from both a material and electrical perspective, successfully demonstrating their suitability as detection interfaces for ultra-stable biosensing applications. KRAS and MUC1 target molecules were successfully assayed in diluted human blood serum with the 3D sensing gate functionalized with b-KRAS and anti-MUC1, achieving a LOD of 10 zM and 40 zM, respectively. Those limits of detection correspond to 1 ± 1 molecules of KRAS and 2 ± 1 molecules of MUC1 in 100 μL sample volume of human blood serum. By efficiently combining organic electronics and 3D printing processes, the 3D SiMoT field-effect sensing system adds tremendous control over the biosensor stability, representing an exceptionally compelling technology to enable noninvasive and cost-effective early diagnosis of life-threatening progressive diseases. As a future perspective, the 3D SiMoT technology might also be expanded into a fully printed electronic array, capable of multiplexing of several biomarkers directly into a peripheral body fluid, such as blood. This could open the door to widespread practice of liquid biopsy for the early diagnosis of high-grade pancreatic mucinous cysts, supporting and possibly improving existing diagnostic practices.

## Data Availability

The data presented in this study are openly available in https://ida.fairdata.fi/. Repository Research data storage service IDA (ida.fairdata.fi).

## References

[1] Finoulst I, Pinkse M, Van Dongen W, Verhaert P. Sample preparation techniques for the untargeted LC-MS-based discovery of peptides in complex biological matrices. J Biomed Biotechnol [Internet]. 2011;2011:245291. Available from: 10.1155/2011/245291 10.1155/2011/245291 PMC3238806 22203783

[2] Carrato A, Falcone A, Ducreux M, Valle JW, Parnaby A, Djazouli K (2015). A Systematic Review of the Burden of Pancreatic Cancer in Europe: Real-World Impact on Survival, Quality of Life and Costs. J Gastrointest Cancer..

[3] Rahib L, Smith BD, Aizenberg R, Rosenzweig AB, Fleshman JM, Matrisian LM (2014). Projecting cancer incidence and deaths to 2030: the unexpected burden of thyroid, liver, and pancreas cancers in the United States. Cancer Res..

[4] Siegel RL, Miller KD, Jemal A (2017). Cancer Statistics, 2017. CA Cancer J Clin..

[5] Malats N, Katsila T, Patrinos GP (2017). Cancer Genomics and Public Health. Public Health Genomics [Internet]..

[6] Basturk O, Hong S-M, Wood LD, Adsay NV, Albores-Saavedra J, Biankin AV (2015). A Revised Classification System and Recommendations From the Baltimore Consensus Meeting for Neoplastic Precursor Lesions in the Pancreas. Am J Surg Pathol..

[7] European evidence-based guidelines on pancreatic cystic neoplasms (2018). Gut..

[8] Tanaka M, Fernández-del Castillo C, Adsay V, Chari S, Falconi M, Jang J-Y (2012). International consensus guidelines 2012 for the management of IPMN and MCN of the pancreas. Pancreatol Off J Int Assoc Pancreatol..

[9] Singhi AD, McGrath K, Brand RE, Khalid A, Zeh HJ, Chennat JS, et al. Preoperative next-generation sequencing of pancreatic cyst fluid is highly accurate in cyst classification and detection of advanced neoplasia. Gut Publ Online First. 2017;2131–41. 10.1136/gutjnl-2016-313586 PMC6241612 28970292

[10] Macchia E, Sarcina L, Driescher C, Gounani Z, Tewari A, Osterbacka R (2021). Single-molecule Bioelectronic label-free assay of both protein and genomic markers of pancreatic mucinous cysts’ in whole blood serum. Adv Electron Mater [Internet].

[11] Klöppel G, Basturk O, Schlitter AM, Konukiewitz B, Esposito I (2014). Intraductal neoplasms of the pancreas. Semin Diagn Pathol..

[12] Rissin DM, Kan CW, Campbell TG, Howes SC, Fournier DR, Song L (2010). Single-molecule enzyme-linked immunosorbent assay detects serum proteins at subfemtomolar concentrations. Nat Biotechnol..

[13] Walt DR (2013). Optical methods for single molecule detection and analysis. Anal Chem..

[14] Tobos CI, Kim S, Rissin DM, Johnson JM, Douglas S, Yan S (2019). Sensitivity and binding kinetics of an ultra-sensitive chemiluminescent enzyme-linked immunosorbent assay at arrays of antibodies. J Immunol Methods [Internet]..

[15] Moody MD, Arsdell SW Van, Murphy KP, Orencole SF, Burns C. Array-Based ELISAs for High-Throughput Analysis of Human Cytokines. Biotechniques [Internet]. 2001;31(1):186–194. Available from: 10.2144/01311dd03 10.2144/01311dd03 11464511

[16] Tobos CI, Sheehan AJ, Duffy DC, Rissin DM. Customizable Multiplex Antibody Array Immunoassays with Attomolar Sensitivities. Anal Chem [Internet]. 2020;92(7):5613–5619. Available from: 10.1021/acs.analchem.0c00631 10.1021/acs.analchem.0c00631 32122115

[17] Cho B, Lee H-H, Shin J-K, Murata M, Ohuchida K, Hashizume M (2012). Detection of pancreatic cancer cells (suit-2) using an fet-based biosensor with an extended au gate. Biomed Eng Appl Basis Commun [Internet]..

[18] Yousefi M, Dehghani S, Nosrati R, Zare H, Evazalipour M, Mosafer J (2019). Aptasensors as a new sensing technology developed for the detection of MUC1 mucin: A review. Biosens Bioelectron..

[19] Macchia E, Manoli K, Di Franco C, Picca RA, Österbacka R, Palazzo G (2020). Organic Field-Effect Transistor Platform for Label-Free, Single-Molecule Detection of Genomic Biomarkers. ACS Sensors..

[20] Macchia E, Manoli K, Holzer B, Di Franco C, Picca RA, Cioffi N (2019). Selective single-molecule analytical detection of C-reactive protein in saliva with an organic transistor. Anal Bioanal Chem..

[21] Macchia E, Sarcina L, Picca RA, Manoli K, Di Franco C, Scamarcio G (2020). Ultra-low HIV-1 p24 detection limits with a bioelectronic sensor. Anal Bioanal Chem..

[22] Macchia E, Tiwari A, Manoli K, Holzer B, Ditaranto N, Picca RA (2019). Label-Free and Selective Single-Molecule Bioelectronic Sensing with a Millimeter-Wide Self-Assembled Monolayer of Anti-Immunoglobulins. Chem Mater..

[23] Macchia E, Manoli K, Holzer B, Di Franco C, Ghittorelli M, Torricelli F (2018). Single-molecule detection with a millimetre-sized transistor. Nat Commun [Internet].

[24] Blasi D, Viola F, Modena F, Luukkonen A, Macchia E, Picca RA (2020). Printed, cost-effective and stable poly(3-hexylthiophene) electrolyte-gated field-effect transistors. J Mater Chem C..

[25] Sarcina L, Torsi L, Picca RA, Manoli K, Macchia E (2020). Assessment of gold bio-functionalization for wide-interface biosensing platforms. Sensors (Switzerland)..

[26] Macchia E, Torricelli F, Bollella P, Sarcina L, Tricase A, Di Franco C (2022). Large-Area Interfaces for Single-Molecule Label-free Bioelectronic Detection. Chem Rev [Internet]..

[27] Chu CH, Sarangadharan I, Regmi A, Chen YW, Hsu CP, Chang WH (2017). Beyond the Debye length in high ionic strength solution: Direct protein detection with field-effect transistors (FETs) in human serum. Sci Rep..

[28] Park SJ, Kwon OS, Lee SH, Song HS, Park TH, Jang J (2012). Ultrasensitive Flexible Graphene Based Field-Effect Transistor (FET)-Type Bioelectronic Nose. Nano Lett [Internet]..

[29] Kim DJ, Park HC, Sohn IY, Jung JH, Yoon OJ, Park JS (2013). Electrical graphene aptasensor for ultra-sensitive detection of anthrax toxin with amplified signal transduction. Small..

[30] Macchia E, De CL, Torricelli F, Di FC, Di FC, Torsi PL, et al. Why a diffusing single-molecule can be detected in few minutes by a large capturing bioelectronic interface. Arxiv Cornell Univ [Internet]. 2022. 10.48550/arXiv.2202.00949. 10.1002/advs.202104381 PMC9284160 35522000

[31] Tang Y, Memaugh R, Zeng X (2006). Nonregeneration protocol for surface plasmon resonance: Study of high-affinity interaction with high-density biosensors. Anal Chem..

[32] Magagnin L, Maboudian R, Carraro C (2002). Gold Deposition by Galvanic Displacement on Semiconductor Surfaces: Effect of Substrate on Adhesion. J Phys Chem B [Internet]..

[33] Sarcina L, Mangiatordi GF, Torricelli F, Bollella P, Gounani Z, Österbacka R (2021). Surface Plasmon Resonance Assay for Label-Free and Selective Detection of HIV-1 p24 Protein. Biosensors..

[34] Kretschmann E, Raether H (1968). Radiative Decay of Non Radiative Surface Plasmons Excited by Light. Zeitschrift fur Naturforsch - Sect A J Phys Sci..

[35] Miyazaki CM, Shimizu FM, Ferreira M. 6 - Surface Plasmon Resonance (SPR) for Sensors and Biosensors. In: Da Róz AL, Ferreira M, de Lima Leite F, Oliveira ONBT-NT, editors. Micro and Nano Technologies [Internet]. 2017. William Andrew Publishing. p. 183–200. Available from: https://www.sciencedirect.com/science/article/pii/B9780323497787000060

[36] Homola J (2003). Present and future of surface plasmon resonance biosensors. Anal Bioanal Chem..

[37] Moran KLM, Lemass D, O’Kennedy R. Surface plasmon resonance-based immunoassays: Approaches, performance, and applications. In: Handbook of Immunoassay Technologies: Approaches, Performances, and Applications. Elsevier; 2018. p. 129–56.

[38] Zhao H, Brown PH, Schuck P (2011). On the distribution of protein refractive index increments. Biophys J [Internet]..

[39] Sam E, Mehdi J, Chaitanya G, Shuai C, W. DR, T. HR. Tunable control of antibody immobilization using electric field. Proc Natl Acad Sci [Internet]. 2015;112(7):1995–9. Available from: 10.1073/pnas.1424592112 10.1073/pnas.1424592112 PMC4343132 25650429

[40] Bobbert PA, Sharma A, Mathijssen SGJ, Kemerink M, de Leeuw DM (2012). Operational Stability of Organic Field-Effect Transistors. Adv Mater [Internet].

[41] Thompson M, Ellison SLR, Wood R (2002). Harmonized guidelines for single-laboratory validation of methods of analysis (IUPAC Technical Report). Pure Appl Chem..

